# Eineinhalb Jahre E-Scooter – Zwischenbilanz in Hamburg: Teil 1: alkoholbedingte Auffälligkeiten im Straßenverkehr

**DOI:** 10.1007/s00194-022-00601-0

**Published:** 2023-01-05

**Authors:** Antonia Kähler, Klaus Püschel, Benjamin Ondruschka, Alexander Müller, Stefanie Iwersen-Bergmann, Jan-Peter Sperhake, Axel Heinemann, Antonia Fitzek

**Affiliations:** Institut für Rechtsmedizin Hamburg, Universitätsklinikum Hamburg-Eppendorf, Butenfeld 34, 22529 Hamburg, Deutschland

**Keywords:** E-Scooter, Electric Scooter, Unfall, Verkehrsdelikt, Alkohol, E-scooter, Electric scooter, Accident, Traffic offence, Alcohol

## Abstract

**Hintergrund:**

Seitdem im Juni 2019 Elektro-Scooter als urbanes Fortbewegungsmittel in Hamburg eingeführt wurden, wurde eine hohe Anzahl von Verstößen gegen die geltenden Gesetze im Straßenverkehr (StVG, StGB) bezüglich Alkoholkonsums durch E‑Scooter-Fahrer festgestellt.

**Ziel der Arbeit:**

Diese Studie hat zum Ziel, einen Überblick über Gefährdung des Straßenverkehrs von E‑Scooter-Fahrern unter Alkoholeinfluss zu erlangen, deren Relevanz hinsichtlich anderer Verkehrsteilnehmergruppen aufzuzeigen und eine erste Zwischenbilanz in Bezug auf deren Frequenz nach eineinhalb Jahren zu ziehen.

**Material und Methoden:**

Die Daten der durch das Institut für Rechtsmedizin des Universitätsklinikums Hamburg-Eppendorf zwischen dem 15.06.2019 und dem 31.12.2020 hinsichtlich ihrer Blutalkoholkonzentration untersuchten alkoholisierten E‑Scooter-Fahrer (*n* = 342) wurden bezüglich ihrer demografischen Informationen sowie der ärztlichen Untersuchungsergebnisse retrospektiv ausgewertet und in den Kontext mit der Gesamtzahl der Verstöße gegen die Gesetze im Straßenverkehr mit anschließender Blutalkoholmessung gebracht.

**Ergebnisse:**

Insgesamt wurden 9,6 % der Gesamtzahl der Verstöße gegen die Gesetze im Straßenverkehr in Verbindung mit anschließender Bestimmung der Blutalkoholkonzentration wurden von E‑Scooter-Fahrern verübt. 87,7 % der Untersuchten waren männlich. Die Blutalkoholkonzentration lag bei 76,9 % der Untersuchten über der für eine absolute Fahruntüchtigkeit beim Benutzen eines Pkw gültigen Grenze von 1,10 ‰. Eine Häufung der Fälle war v. a. in den Nachtstunden sowie an den Wochenenden auffällig.

Aufgrund unpräziser Aufzeichnungen ist eine gewisse Dunkelziffer von E‑Scooter-Vorfällen unter den nicht näher bezeichneten Kraftfahrzeugen anzunehmen.

**Diskussion:**

Da E‑Scooter-Fahrer einen bedeutenden Anteil unter den alkoholisierten Verkehrsteilnehmern einnehmen und die Vorfälle meist nachts und am Wochenende stattfanden, scheinen eine vermehrte Aufklärung über Gefahren bei Nutzung der E‑Scooter unter Alkoholeinfluss und ggf. ein Fahrverbot zu diesen Zeiten sinnvoll zu sein.

## Abstract (en)

**Background:**

Since electric scooters were introduced as an urban means of transportation in Hamburg in June 2019, a high number of violations of the current laws regarding alcohol consumption by e‑scooter riders have been recorded.

**Objective:**

The aim of this study is to obtain an overview of traffic offences committed by e‑scooter drivers under the influence of alcohol, to classify their relevance in relation to other road user groups, and to draw a first interim balance with respect to their frequency after 1.5 years.

**Material and methods:**

The data of all e‑scooter drivers (*n* = 342) examined concerning their blood alcohol values analyzed at the Institute of Legal Medicine of the University Medical Centre Hamburg-Eppendorf between 15.06.2019 and 31.12.2020 were retrospectively evaluated with respect to their demographic information and the medical examination results. These were brought into context with the total number of offences against the road traffic regulations with subsequent blood alcohol measurement.

**Results:**

9.6% of the total number of offences against the road traffic regulations in connection with subsequent determination of the blood alcohol concentration were committed by e‑scooter drivers. 87.7% of those examined were male. The blood alcohol concentration was above the limit of 1.10 ‰ for absolute driving incapacity when using a passenger car in 76.9% of those examined. An accumulation of cases was particularly noticeable at night and at weekends.

Due to imprecise records, a certain number of unreported e‑scooter incidents can be assumed among the unspecified motor vehicles.

**Conclusion:**

As e‑scooter drivers make up a considerable proportion of drunken road users and the accidents mostly occur at night and at weekends, increased education and, if necessary, a driving ban at these times would seem to make sense.

## Einleitung

Seit der Einführung der sog. Elektro-Scooter durch Verleihservices in Hamburg am 21.06.2019 sind diese Gegenstand wissenschaftlicher und öffentlicher Diskussionen. In den Medien wurden mehrfach Berichte über ein hohes Aufkommen alkoholisierter E‑Scooter-Fahrer veröffentlicht, wie beispielsweise im Rahmen des Oktoberfestes 2019 [1–3]. Medizinische Aufsätze beschrieben v. a. Unfälle, ihre Mechanismen und die daraus resultierenden Verletzungen [4–7] sowie allgemeine Gesundheitsbeeinflussungen durch den Gebrauch von E‑Scootern [8]. Hierbei fiel auf, dass häufig ein nichtunwesentlicher Anteil der untersuchten, verunfallten E‑Scooter-Fahrer vor dem Unfall Alkohol konsumiert hatte [4, 9].

Bezüglich der Alkoholgrenzwerte gelten beim Fahren von E‑Scootern dieselben Gesetze wie bei Kraftfahrzeugführern in Deutschland [10]. Es ist bekannt, dass das Fahren unter Einfluss von Alkohol ohne alkoholbedingte Auffälligkeit, bei einer Blutalkoholkonzentration von 0,50–1,09 ‰ eine Ordnungswidrigkeit darstellt [11]. Das Führen eines Fahrzeuges mit einer Blutalkoholkonzentration von 1,10 ‰ oder mehr sowie das Fahren mit alkoholbedingten Ausfallerscheinungen, wie beispielsweise Orientierungsstörungen, Koordinations- und Konzentrationsstörungen bei einer Blutalkoholkonzentration von mehr als 0,3 ‰ [11] und die hierdurch bedingte Gefährdung des Straßenverkehrs stellen eine Straftat dar. Für Fahrer jünger als 21 Jahre und für Personen in der Probezeit ihres Führerscheins gelten 0,00 ‰ Blutalkoholkonzentration als Grenzwert für die Nutzung eines Elektrokleinstfahrzeuges [12].

Im Rahmen des mobilen Beweismittelsicherungsdienstes im Auftrag der Polizei Hamburg werden durch Ärzte des Instituts für Rechtsmedizin (IfR) des Universitätsklinikums Hamburg-Eppendorf (UKE) Blutentnahmen bei Verdacht der Gefährdung des Straßenverkehrs durch Verkehrsteilnehmer durchgeführt, zu denen seit Mitte 2019 auch die E‑Scooter-Fahrer gehören. Für die vorliegende Studie wurden für einen Zeitraum von 19 Monaten retrospektiv alle im IfR untersuchten Fälle ab Zulassung der E‑Scooter im Juni 2019 bis Dezember 2020 im Hinblick auf die definierten Alkoholgrenzwerte und stattgehabte Unfälle ausgewertet.

Ziel dieser Studie war, eine Übersicht über die Frequenz einer bestehenden Alkoholisierung unter E‑Scooter-Fahrern im Hamburger Stadtgebiet zu erlangen.

## Material und Methodik

Das IfR Hamburg ist zuständig für alle durch die Ermittlungsbehörden im Hamburger Stadtgebiet angeordneten Blutentnahmen und die Bestimmung der in den Blutproben enthaltenen Blutalkoholkonzentrationen. Die zuständigen Ärzte führen die Blutentnahmen durch, wenn bei Verkehrsdelikten der Verdacht auf eine Alkoholisierung besteht und/oder der Alkoholkonsum durch einen Atemalkoholtest der Exspirationsluft bestätigt ist. Der Einzugsbereich hierfür ist das gesamte Stadtgebiet Hamburgs, ergänzt durch wenige außerhalb des Stadtgebietes gelegene Polizeikommissariate.

Für diese Arbeit wurden retrospektiv zunächst alle Blutentnahmen zur Bestimmung der Blutalkoholkonzentration in Verbindung mit einer Gefährdung des Straßenverkehrs zwischen dem 15.06.2019 und dem 31.12.2020 dahingehend händisch untersucht, ob es sich um E‑Scooter-Fahrer handelte oder nicht. Eingeschlossen in diese Studie wurden somit alle Fälle, die durch die Polizei auf den Antragsformularen für die Blutentnahme als „E(lektro)-Kleinstfahrzeug“, „E(lektro)-Roller“, „E(lektro)-Scooter“, „E-Tretroller“, „eKF“, „eKl(K)fz“, „elektronisch betriebener Roller“ und „elektrischer Kleinroller“ bezeichnet wurden. Wenn dies zutraf, wurden Datum und Uhrzeit des Deliktes, die Bezeichnung des Fahrzeuges, das Geschlecht, die gesprochene Sprache des Delinquenten und die aus jeweils 4 Einzelbestimmungen ermittelte mittlere Blutalkoholkonzentration (inklusive minimale und maximale Werte) festgehalten. Zusätzlich wurden das meldende Polizeikommissariat und das Ergebnis der orientierenden ärztlichen Untersuchung im Sinne einer nichtmerklichen, leichten, mittelgradigen oder hochgradigen (alkoholbedingten) Einschränkung erfasst.

Die statistische Analyse erfolgte deskriptiv mit Microsoft Excel (Version 16.16, Microsoft Corporation, Redmond, USA). Die Variablen wurden als Prozentsätze und Absolutzahlen beschrieben. Zusätzlich wurde der Pearson-Chi-Quadrat-Test zum Test auf einen statistisch signifikanten Zusammenhang der kategorialen Variablen durchgeführt. Diese Testung und die grafische Darstellung der Ergebnisse erfolgten mit der Statistiksoftware GraphPad Prism® (Version 8.0, GraphPad Software Inc., La Jolla, USA).

## Ergebnisse

Von insgesamt 3567 Verkehrsdelikten, bei denen eine Bestimmung der Blutalkoholkonzentration vorgenommen wurde, wurden 342 (9,6 %) Elektro-Scooter-Fälle identifiziert. Im Vergleich gab es 1612 Pkw-Fahrer (45,2 %), 394 Radfahrer (11,1 %), 38 Lkw-Fahrer (1,1 %), 30 Kleinkrafträder (0,8 %), 25 Bootsführer (0,7 %), 14 Krads (0,4 %), 4 Fußgänger (0,1 %) sowie jeweils ein Hoverboard, Quad und Flugzeug (je 0,03 %). Zudem fehlte in 1105 Fällen (31,0 %) eine nähere Beschreibung des Fahrzeuges.

Von den 342 Elektro-Scooter-Fahrern waren 300 (87,7 %) männlich und 42 (12,3 %) weiblich (*p* = 0,0439).

287 (83,9 %) der erfassten Personen waren deutschsprachig, und 50 (14,6 %) sprachen eine andere Sprache. Bei 5 Beschuldigten war anhand der Daten nicht ersichtlich, ob sie der deutschen Sprache mächtig waren oder nicht (1,5 %) (Tab. 1).

**Table 1:** 

Beschreibung	Kategorisierung	Absolute Werte	Relative Werte (in %)
Gemessener Wert der Blutalkoholkonzentration zum Tatzeitpunkt	< 0,3 ‰	3	0,9
	0,3–0,5 ‰	4	1,2
	0,5–1,1 ‰	69	20,2
	> 1,1 ‰	266	77,8
Geschlecht	Männlich	300	87,7
	Weiblich	42	12,3
Sprache	Deutsch	287	83,9
	Englisch	27	7,9
	Französisch	1	0,3
	Andere Sprache	22	6,4
	Keine Angabe	5	1,5
Einschränkung laut Untersuchungsergebnis	Nicht merkbar	40	11,7
	Leicht	176	51,5
	Mittelgradig	78	22,8
	Hochgradig	3	0,9
	Keine Angabe	45	13,2
Wochentag	Montag	29	8,5
	Dienstag	23	6,7
	Mittwoch	27	7,9
	Donnerstag	36	10,5
	Freitag	60	17,5
	Samstag	97	28,4
	Sonntag	70	20,5
Uhrzeit	00:00–05:00	259	75,7
	06:00–11:00	21	6,1
	12:00–17:00	9	2,6
	18:00–23:00	53	15,6

### Der Mittelwert der gemessenen Blutalkoholkonzentration aller E-Scooter Fahrer lag > 1,1 ‰

Der Mittelwert der gemessenen Blutalkoholkonzentration aller E‑Scooter-Fahrer lag bei 1,3 ‰ (Standardabweichung ± 0,41 ‰), der Median bei 1,2 (Verteilung: 0,00–2,61). Der größte Teil der Blutalkoholwerte lag mit 266 Fällen (77,8 %) über 1,10 ‰. 69 (20,2 %) der Werte lagen im Bereich zwischen 0,5 ‰ und 1,1 ‰. Lediglich in 3 Fällen (0,9 %) lag die Blutalkoholkonzentration unter 0,3 ‰, nur in 4 Fällen (1,2 %) lag sie zwischen 0,3 ‰ und 0,5 ‰. Im Vergleich ergab die orientierende ärztliche Untersuchung im Rahmen der Blutentnahme in 40 Fällen keine alkoholbedingten Ausfallerscheinungen (11,7 %), in 176 Fällen (51,5 %) eine leichte, in 78 Fällen (22,8 %) eine mittelgradige und in 3 Fällen (0,9 %) eine hochgradige alkoholbedingte Einschränkung der Probanden. In 45 Fällen (13,2 %) wurden hierzu keine gesonderten Feststellungen getroffen (Tab. 1). 71 der E‑Scooter-Fahrer (26,6 %) mit einer BAK > 1,10 ‰ wurden mit alkoholbedingten Ausfallerscheinungen auffällig, im Vergleich zu 6 (9 %) mit 0,5–1,1 ‰, einem (20,0 %) mit 0,3–0,5 ‰ und keinem (0,0 %) mit weniger als 0,3 ‰. Damit zeigt sich ein signifikanter Unterschied in der Häufigkeit des Auftretens von Ausfallerscheinungen < 1,10 ‰ und > 1,10 ‰ (*p* = 0,0071).

### Allgemeine Häufung der Vergehen in der Innenstadt in den Sommermonaten, am Wochenende und zwischen 22 Uhr und 6 Uhr

**Figure 1:**
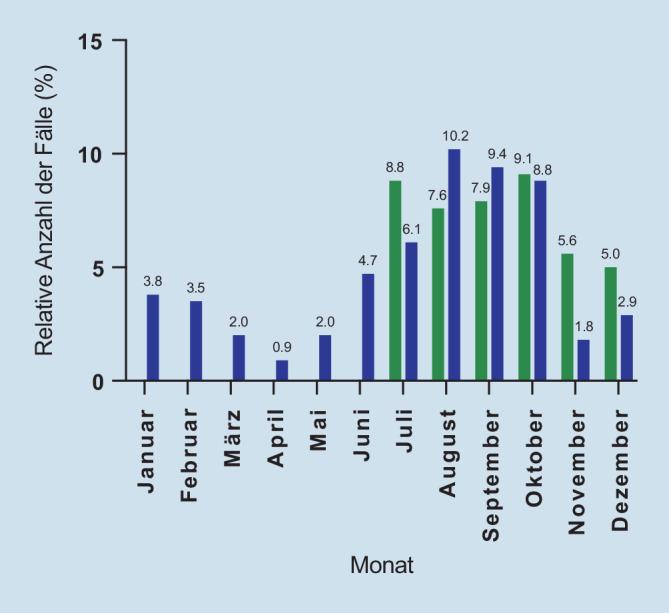

Von Juli bis Oktober 2019 wurden mit 114 Fällen 33,3 %, im gleichen Zeitraum 2020 mit 118 Fällen 34,5 % und somit insgesamt in den Monaten Juli bis Oktober mit 232 Fällen 67,8 % aller registrierter Fahrten mit einem E‑Scooter unter Alkoholeinfluss begangen. Die niedrigste Vorfallfrequenz zeigte sich zwischen März 2020 und Mai 2020 mit durchschnittlich 5,7 Delikten (1,7 %) pro Monat sowie im November und im Dezember 2020 mit durchschnittlich 8 Vorfällen (2,3 %). Räumlich waren die meldenden Polizeikommissariate zum größten Teil innerstädtisch oder in Innenstadtnähe der Stadt Hamburg angesiedelt. 227 (66,4 %) der Delikte traten am Wochenende zwischen Freitag und Sonntag auf. Die wenigsten Fälle fanden sich mit 23 (6,7 %) dienstags, die meisten mit 97 (28,4 %) am Samstag. Bezüglich der deliktrelevanten Uhrzeiten zeichnete sich ein klarer Trend ab: In 259 von 342 Fällen (75,7 %) wurden die E‑Scooter unter Alkoholeinfluss zwischen Mitternacht und 05:00 Uhr morgens benutzt. Zwischen 06:00 und 23:59 Uhr gab es insgesamt 83 (24,3 %) Vorkommnisse (Tab. 1, Abb. 1). Es zeigten sich damit signifikant häufiger Fälle am Wochenende und in den Nachtstunden (*p* = 0,0089).

## Diskussion

Ziel dieser Studie war, eine Übersicht über die Häufigkeit polizeibekannter Verkehrsdelikte von unter Alkoholeinfluss stehenden Nutzern von E‑Scootern im Hamburger Stadtgebiet nach 1,5 Jahren regionaler Nutzungszeit zu erlangen.

Es wurde dabei gezeigt, dass die Blutalkoholkonzentration bei den meisten untersuchten E‑Scooter-Fahrern über 1,10 ‰ lag. Das frequente Überschreiten des für die Rechtsprechung elementaren Grenzwertes der absoluten Fahruntüchtigkeit kann weitreichende Konsequenzen, von Geldstrafen über den Entzug der Fahrerlaubnis bis hin zu Freiheitsstrafen, nach sich ziehen. Auf dem Münchner Oktoberfest in 2019 wurden beispielsweise 254 alkoholisierten E‑Scooter-Fahrern der Führerschein entzogen [3]. Die präsentierten Ergebnisse lassen eine mangelnde Kenntnis oder Desinteresse an der Rechtslage bezüglich der Alkoholgrenzwerte bei E‑Scooter-Fahrern, eine Unterschätzung der für deren sicheren Betrieb notwendigen fein- und grobmotorischen Fähigkeiten und nicht zuletzt auch eine Bagatellisierung der mit Alkoholeinfluss grundsätzlich erhöhten Unfallwahrscheinlichkeit vermuten. Bereits in einer Münchner Studie wurden über den Promille-Grenzwert erhöhte Blutalkoholkonzentrationen bei über 80 % der E‑Scooter-Fahrer festgestellt [13]. Auch der allgemeingültige Trend, dass mehr und intensivere Ausfallerscheinungen bei höheren BAK-Werten zu erwarten sind, ließ sich statistisch bestätigen.

Wie auch in bisher zu Unfällen von E‑Scooter-Fahrern publizierten Studien war der größte Teil der alkoholisierten Fahrer männlich, wobei dies für Hamburg mit 87,7 % noch einmal deutlicher abzugrenzen war als zuvor zwischen 56,7 und 73,9 % [4, 9, 14].

Die auch in Frankfurt a. M. erhöhte Fallzahl in den Sommermonaten lässt sich auf die besseren Witterungsverhältnisse und auch auf die höhere Verfügbarkeit der Elektroroller zurückführen, welche in den Wintermonaten durch die Anbieter aus ökonomischen Gründen vermehrt aus dem Umlauf entfernt wurden [15]. Gleichzeitig korrelieren die ausgesprochen niedrigen Fallzahlen in den Monaten März bis Mai und November sowie Dezember 2020 mit den Wellen der SARS-CoV‑2-Pandemie (schweres-akutes-Atemwegssyndrom-Coronavirus Typ 2) in Hamburg [16]. Zu diesen Intervallen war das öffentliche Leben in Deutschland jeweils stark durch Hygienemaßnahmen und Pandemieeindämmungen eingeschränkt und der Bedarf an flexiblen Transportmitteln wie E‑Scootern weniger vorhanden, was auch durch Störmann et al. und Mair et al. beobachtet werden konnte [15, 17].

Die Tatsache, dass ein sehr großer Anteil der ausgewerteten Verkehrsdelikte durch das alkoholisierte Fahren von E‑Scootern an den Wochentagen Freitag bis Sonntag und v. a. in den späten Abend- und frühen Morgenstunden begangen wurde, könnte darauf hinweisen, dass E‑Scooter als praktische Alternative zu anderen Verkehrsmitteln angesehen werden, um sich zeitsparend und flexibel auch in angetrunkenem Zustand im öffentlichen Verkehr zu bewegen. Auch bei anderen Studien fanden die Fahrten unter Alkoholeinfluss in drei Viertel der Fälle in den Nachtstunden statt [14]. Leicht konträr berichteten Trivedi et al. bei nur vereinzelten Fällen alkoholisierter Fahrten (4,8 %) von Auffälligkeiten v. a. zwischen 15 und 23 Uhr [9]. Es wurde ein nächtliches Fahrverbot für E‑Scooter vorgeschlagen [18], welches nach der dieser Studie zugrunde liegenden Datenlage eine mögliche Lösung zur Verringerung der Verkehrsdelikte unter Alkoholeinfluss mit Beteiligung von E‑Scooter-Fahrern darstellen könnte.

Es kann in Anbetracht der häufig verwendeten Fremdsprachen davon ausgegangen werden, dass E‑Scooter auch bei Touristen und ausländischen Personen ein beliebtes Verkehrsmittel darstellen, wie zuvor bereits in Kalifornien beobachtet [19]. Daraus ergibt sich eine internationale Problematik bezüglich ländereigener Regelungen für die Nutzung von E‑Scootern und die Nichtkenntnis oder Nichtbeachtung der deutschen Gesetzeslage [15].

## Limitationen

Diese Studie unterliegt einigen Limitationen, welche im Folgenden näher beleuchtet werden.

Zum einen könnte durch nichteinheitliche Dokumentation seitens der Polizei auf den entsprechenden Formularen Unklarheiten darüber entstehen, welche Fahrzeuge konkret gemeint sind. Es ist denkbar, dass sich unter den nicht näher bezeichneten Verkehrsteilnehmern, welche 31,0 % der Gesamtanzahl ausmachten, noch weitere Fälle von E‑Scooter-Fahrern befinden, welche nicht in die Statistik aufgenommen werden konnten. Es wird eine einheitliche Bezeichnung für die Dokumentation angeraten, um wissenschaftliche Auswertungen zu präzisieren.

Zum anderen bleibt verborgen, ob Ort- und Zeithäufungen auch durch einsatztaktische Vorgaben der Polizei geprägt sind, was eine Dunkelziffer in anderen Stadtvierteln und zu anderen Uhrzeiten vermuten ließe. Die innenstadtnahe hohe Frequenz ist auch mit der grundsätzlich höheren zentralen Verfügbarkeit der Leihgeräte zu verbinden.

In den vorliegenden Daten waren keine demografischen Details der Probanden vermerkt, weswegen eine Grundlage für eine gezielter an bestimmte Bevölkerungsgruppen gerichtete Prävention der Nutzung von E‑Scootern unter Alkoholeinfluss aus diesen Daten nicht abzuleiten ist. Es ist durchaus wahrscheinlich, dass durch den Einschluss der Corona-Pandemie in die Erhebungszeit eine die Datenlage relevant beeinflussende Varianz in der Nutzung der E‑Scooter vorliegen könnte.

Die analysierten Messwerte und ärztlichen Einschätzungen treffen für den Zeitpunkt der Blutentnahme zu. Es ist für das hier beschriebene Kollektiv davon auszugehen, dass diese aufgrund der Organisationsstruktur des mobilen Beweismittelsicherungsdiensts in Hamburg jeweils zeitnah zur Trunkenheitsfahrt durchgeführt werden konnte. Eine längere Wartezeit bei hohem Fallaufkommen für den diensthabenden Arzt kann aber retrospektiv nicht ausgeschlossen werden, somit ist eine noch höhere Blutalkoholkonzentration zum Deliktzeitpunkt im Einzelfall vorstellbar.

Ein möglicher Mischkonsum mit anderen Rauschmitteln konnte nicht in diese Studie eingeschlossen werden, da lediglich die Daten der Untersuchungen auf Ethanol zur Verfügung standen.

Zuletzt sei darauf verwiesen, dass die Anordnung einer Blutentnahme regelhaft nur dann geschieht, wenn seitens der Ermittlungsbehörden der berechtigte Anhaltspunkt für eine bestehende Alkoholisierung existiert. Insofern verwundert nicht, dass in fast allen Einzelfällen auch eine analytisch nachweisbare Alkoholkonzentration in den Blutproben nachgewiesen werden konnte. Durch die Studie kann somit nicht beantwortet werden, wie viele der einen E‑Scooter nutzenden Personen dabei alkoholisiert sind.

Trotz dieser Einschränkungen konnte diese Studie einen Einblick in die Problematik alkoholisierter E‑Scooter-Fahrer – am Beispiel Hamburg – geben, diese in einen Kontext von Nutzungszeiten und -charakteristika stellen und Ansatzpunkte für Prävention und Eindämmung liefern.

## Ausblick

Da voraussichtlich auch weiterhin ein hohes und erwartbar noch größer werdendes Angebot und eine einfache Verfügbarkeit der E‑Scooter bestehen bleiben wird, wäre eine Sensibilisierung der Nutzer für die deutsche Gesetzeslage und die definierten Grenzwerte für alkoholisiertes Fahren mit E‑Scootern sinnvoll. Praktikabel erscheint eine Integration dieser Informationen in denen zur Buchung der Roller verwendeten Handyapplikationen und an den E‑Scootern selbst in Form von Warnhinweisen/Aufklebern. Diese Empfehlungen wären nicht nur eine Hilfestellung für die jeweiligen Nutzer zur Vermeidung hoher Geldstrafen, den Führerscheinentzug oder ggf. sogar eine Freiheitsstrafe. Genauso relevant ist der Schutz anderer Verkehrsteilnehmer, die durch die alkoholisierte Fahrweise gefährdet werden könnten. Hinzu kommen Personen aus anderen Ländern, in denen evtl. andere Regelungen bezüglich der E‑Scooter gelten, welche nach Möglichkeit über die hiesigen Gesetze informiert zu sein haben.

Einige Anbieter von E‑Scooter-Sharing fordern die Nutzer ihrer Apps bereits auf, die Information zur Kenntnis zu nehmen, dass die alkoholisierte Nutzung der Fahrzeuge rechtliche Konsequenzen haben kann. Der Verleihservice LIME zeigt beispielsweise beim Öffnen der App eine Mitteilung an, welche eine Bestätigung erfordert, dass nicht unter Alkoholeinfluss gefahren werden sollte. Diese könnte vor jeder Entleihe, v. a. am Wochenende und nachts angezeigt werden, um eine Prävention zu ermöglichen.

Um auch die Zusammenhänge zwischen der Alkoholisierung, alkoholtypischen Ausfallerscheinungen und verunfallten E‑Scooter-Fahrern näher wissenschaftlich analysieren zu können, wäre eine Zusammenführung der Daten aus dieser Studie mit polizeilichen und klinischen Informationen bezüglich stattgehabter Unfälle sinnvoll.

## Fazit für die Praxis

- E‑Scooter werden ganz offenbar von alkoholisierten, weit überwiegend männlichen Personen als probates Verkehrsmittel im Straßenverkehr wahrgenommen.
- Die Blutalkoholkonzentration bei alkoholisierten E‑Scooter-Fahrern liegt häufig über 1,10 ‰, was strafrechtlich erhebliche Folgen für die Delinquenten haben kann.
- Die Fahrten unter Alkoholeinfluss finden v. a. nachts, am Wochenende und in den Sommermonaten statt.
